# HCSRL: hyperledger composer system for reducing logistics losses in the pharmaceutical product supply chain using a blockchain-based approach

**DOI:** 10.1038/s41598-024-61654-7

**Published:** 2024-06-12

**Authors:** Satyabrata Dash, Umashankar Ghugar, Deepthi Godavarthi, Sachi Nandan Mohanty

**Affiliations:** 1 Department of Computer Science & Engineering, GITAM School of Technology, GITAM (Deemed to Be University), Vishakhapatnam, India; 2 Department of CSE, School of Engineering, OP Jindal University, Raigarh, CG India; 3 School of Computer Science & Engineering (SCOPE), VIT-AP University, Amaravati, Andhra Pradesh India

**Keywords:** Blockchain, Sustainable supply chain, Hyperledger, Performance measurement, Pharmaceutical product supply chain, SCRM, Drug discovery, Health care

## Abstract

Blockchain technology uses a secure and decentralised framework for transaction management and data sharing within supply chains. This is particularly crucial in the pharmaceutical industry, where product authenticity and traceability are paramount. Blockchain plays a pivotal role in preventing product loss and counterfeiting, while simultaneously enhancing transparency and efficiency throughout the supply chain. The research introduces a step-by-step approach to implementing a proof-of-concept (PoC) for Supply Chain Risk Management (SCRM) through blockchain technology. This PoC involves simulating a supply chain process to assess feasibility and measure key performance indicators. Engaging stakeholders and gathering feedback is integral to refining the blockchain-based SCRM system. The study rigorously evaluates the performance of the SCRM blockchain across various test scenarios, featuring differing numbers of organizations and clients. Multiple fabric networks are employed to assess the system’s scalability and performance under diverse conditions. The results of these comprehensive tests inform practical deployment decisions and highlight areas for potential optimization and further development. So this research provides valuable insights into the application of blockchain in pharmaceutical supply chains, offering a roadmap for implementation and improving supply chain security, efficiency, and transparency.

## Introduction

Blockchain can be used to track pharmaceutical products at every step of the supply chain, from manufacturing to distribution, and ultimately to the end-user. Every transaction available in the supply chain process is stored in a tamper-proof and immutable ledger, enabling transparency and accountability. This tracking feature can help prevent losses due to theft, diversion, or counterfeiting, as any unauthorized changes or attempts to modify the data would be detected and flagged. Shipping of raw materials, particularly in the pharmaceutical industry, involves multiple levels and personalities, and is vulnerable to various external factors such as theft or modification. Such events can result in significant losses and pose a threat to human life^1^. In the shipping process Blockchain can also help prevent loss and counterfeiting of pharmaceutical products by providing a secure and decentralized platform for authentication and verification. Each product can be assigned a unique digital identity, which can be verified by all stakeholders in the supply chain, including manufacturers, distributors, and retailers. This verification process ensures that only authentic products are sold to end-users, reducing the risk of loss and harm caused by counterfeit products^2^. Digital evidence plays a crucial role in investigating any illegal activities, especially in complex processes like shipping raw materials. The Shipping Chain of Raw Material (SCRM) is an effective system that maintains the integrity of pharmaceutical products by keeping all the information of interactions with different specification and their related work in online form. This system ensures the preservation of digital evidence throughout the entire lifecycle of the investigation. SCRM logs all the relevant information such as when and how digital evidence of events is recorded and preserved^3^. This transparency in recording and preserving digital evidence can be helpful in any kind of investigation where the integrity and originality of evidence are critical. Blockchain technology is an ideal solution for SCRM as it offers tamper-resistance, data integrity, and transparency, which are critical for recording the integrity of the raw materials throughout the shipping process. By using blockchain, all the records related to the shipping of raw materials can be securely stored in a decentralized database, which cannot be tampered with by any individual or entity^4^. In addition to maintaining the integrity of the raw materials during the shipping process, SCRM also plays a crucial role in digital investigations. By providing an unalterable digital record of all the events related to the shipping of raw materials, SCRM makes it easier to identify any illegal activities that may have occurred during the process^5,6^. So the use of SCRM powered by blockchain technology is essential in maintaining the integrity of raw materials during the shipping process and ensuring the safety and efficacy of pharmaceutical products. It also plays a crucial role in digital investigations by providing a transparent and unalterable digital record of all events related to the shipping process.

Finally the pharmaceutical product supply chain is plagued by various challenges, including counterfeiting, diversion, and theft, leading to significant financial losses and potential health risks for consumers. Motivated by the urgent need to enhance traceability, transparency, and security in this critical industry, the proposed blockchain framework with a proof-of-concept (PoC) in Hyperledger Composer aims to prevent losses by creating an immutable and decentralized ledger that tracks the movement of pharmaceutical products from manufacturing to distribution. This innovative solution harnesses blockchain technology to address the problem of product loss, bolstering the integrity of pharmaceutical supply chains and ultimately safeguarding public health.

The organization of this paper is as follows: Section “Literature review” illustrates the various research work performed in this field; Section “Materials and methods” demonstrates the materials and the methods; Section “Blockchain for pharmaceutical product supply chain” discusses the use of blockchain for the product supply chain required for this study; Section “Shipping process of raw material in SCM” provides the details of the shipping process of the materials; Section “Proposed model” provide simulations results and discussion; Finally, Section “Conclusion” concludes this study.

## Literature review

The manufacturing and distribution of counterfeit drugs present a significant challenge globally, particularly during pandemics when there's an urgent demand for medical treatments. The pharmaceutical industry's robust supply chain system plays a crucial role in combating this issue. However, challenges persist, especially in developing countries with inadequate regulatory oversight and weak enforcement mechanisms that allow criminal networks to exploit vulnerabilities. Counterfeit drugs, intentionally mislabelled or misrepresented, pose serious risks to patients, including ineffectiveness, contamination, and harmful substances. These risks are amplified by the complexity of the pharmaceutical supply chain, involving multiple stages from manufacturing to retail, providing opportunities for counterfeiters to infiltrate the system^7^. Several factors contribute to the prevalence of counterfeit drugs, including the high cost of genuine medicines, insufficient regulatory supervision, and limited public awareness. Addressing these challenges requires a multifaceted approach involving collaboration between government agencies, pharmaceutical firms, healthcare providers, and patients. One promising solution explored by researchers is leveraging blockchain technology for secure pharmaceutical product delivery. Blockchain offers transparency, traceability, and tamper-resistant features that can enhance supply chain integrity. It enables stakeholders to verify the authenticity and origin of drugs at each stage, reducing the risk of counterfeit products entering the market. So implementing blockchain in the medical product supply chain faces its own challenges, such as technological complexity, interoperability issues, cost considerations, and regulatory compliance^8,9^. Overcoming these hurdles requires coordinated efforts, investment in infrastructure, standards development, and education to ensure effective adoption and integration of blockchain solutions. So while blockchain holds promise in addressing counterfeit drugs and improving supply chain security, tackling the broader challenges of medical product supply chains necessitates a comprehensive strategy involving diverse stakeholders and addressing various technical, regulatory, and awareness-related issues. This literature survey explains some important outlines of various researchers working on the security of pharmaceutical product delivery using blockchain.

The study by Abbasi, Sina, Çiğdem Sıcakyüz, and Babek Erdebilli explores the critical task of designing an effective home healthcare supply chain in the midst of a health crisis. The authors address the pressing need for a well-structured and responsive system to ensure the seamless delivery of healthcare resources to patients in their homes. By integrating logistics and resource allocation strategies, the research provides valuable insights into optimizing the supply chain for home healthcare, ultimately contributing to more efficient and resilient healthcare systems in times of crisis. The study's findings have significant implications for enhancing healthcare delivery, particularly during challenging public health emergencies^9–11^.

Tseng et al.^12^ proposed A blockchain and IoT-based pharma supply chain system that can certainly help to ensure the authenticity of data sources, IoT devices, sensors, locators, and QR codes. This approach involves using distributed ledger technology to store and share information in a secure and transparent manner. With this system, each transaction and event within the supply chain is recorded on a decentralized ledger that cannot be altered or manipulated, ensuring that the data is tamper-proof and trustworthy^13,14^. IoT devices, sensors, and locators can also be used to track the movement of products through the supply chain, providing real-time data on their location and condition. So has the potential to revolutionize the pharmaceutical industry, providing greater transparency, safety, and efficiency in the production and distribution of drugs.

Abbasi et al.^15^ explain about Gcoin Blockchain which is a relatively new blockchain technology that has been developed specifically for use in the pharmaceutical industry. It is based on a combination of a decentralized autonomous organization regulation model and open government, which aims to ensure transparent drug transaction data flow. The decentralized autonomous organization regulation model used by Gcoin Blockchain is also interesting. It allows for greater transparency and accountability in the pharmaceutical product supply chain by creating a system of self-regulation, where all stakeholders have a voice in the decision-making process^16^. This can help to reduce the risk of fraud and counterfeit drugs, as well as improve the overall quality of pharmaceutical products. It is an innovative solution that has the potential to revolutionize the pharmaceutical industry. Its combination of a decentralized autonomous organization regulation model and open government principles, along with its scalability, make it a promising technology for ensuring transparent drug transaction data flow and improving the safety and efficacy of pharmaceutical products.

Hasan et al.^16^ proposed blockchain-based solution that utilizes smart contracts in the Ethereum blockchain to manage transactions between the sender and receiver is an innovative approach to ensuring the safety and security of pharmaceutical products during shipping. By leveraging the capabilities of smart contracts, this solution can automate and streamline the entire supply chain process, from order fulfilment to delivery. This can help to reduce costs, eliminate intermediaries, and provide greater transparency and accountability throughout the process. this solution has the potential to revolutionize the pharmaceutical industry by improving the safety, security, and efficiency of the supply chain. By leveraging the power of blockchain and IoT, it can help to ensure that pharmaceutical products are delivered safely and securely to patients, no matter where they are located^17^.

Shaikh et al.^18^ explained Transparency and traceability are critical elements in any supply chain management system, as they enable companies to monitor and control the distribution of product and services, and ensure compliance with regulations and industry standards. In the past year blockchain technology has emerged as a significant solution for improving transparency and traceability in various industries, including food, pharmaceuticals, consumer electronics, and automobiles. The use of blockchain-based traceability solutions has the potential to transform the supply chain management landscape, by providing greater transparency, traceability, and accountability throughout the process.

In their paper titled "Lightweight-BioV: Blockchain Distributed Ledger Technology (BDLT) for Internet of Vehicles (IoVs)," Laghari et al. explore the innovative use of blockchain technology in the context of Internet of Vehicles (IoVs). The study delves into the development of Lightweight-BioV, a blockchain-based distributed ledger system tailored for IoVs. This technology has the potential to enhance the security, transparency, and data management within the IoV ecosystem. The research sheds light on the significant implications of integrating blockchain into IoVs and could contribute to the evolution and improved functionality of connected vehicle systems^19,20^.

Bocek et al.^20^ provided the solution developed by modum.io is an excellent example of how IoT and blockchain technology can be used to ensure quality control during the shipping of medicines. By measuring the temperature and humidity of medicine containers during shipping using IoT sensors, this solution can detect any deviations from the prescribed conditions, and alert the relevant parties in real-time. This data is stored on a public blockchain architecture provided by Ethereum, ensuring that it is tamper-proof, transparent, and auditable. This makes it easy to track the movement of medicines from the point of origin to the final destination and to identify any issues that may arise during the shipping process. Overall, the modum.io solution is an excellent example of how blockchain technology can be used to solve real-world problems and improve the quality of life for people around the world^21–23^.

The paper, authored by Abbasi and Ahmadi Choukolaei, presents a systematic review of green supply chain network design literature, with a specific focus on carbon policy. The study explores various aspects of green supply chain network design and its relationship with carbon policies. The authors provide a comprehensive analysis of existing research in this domain, shedding light on the critical intersection of sustainability and carbon reduction within supply chain networks. This review article offers valuable insights into the evolving landscape of sustainable supply chain management, emphasizing the significance of environmental policies in shaping contemporary business practices^24^.

The paper by Khan, Shaikh, and Laghari delves into the integration of the Internet of Things (IoT) and multimedia investigation within the context of digital forensics chain-of-custody. It presents a secure approach employing blockchain technology, specifically Hyperledger Sawtooth, to maintain the integrity and authenticity of digital evidence. The study focuses on ensuring a tamper-proof and transparent chain-of-custody process, critical in legal proceedings. By combining IoT and multimedia, this innovative approach enhances the accuracy and reliability of digital forensics, offering a promising solution for maintaining the trustworthiness of evidence in an increasingly digital world. The research contributes to the field of cybercrime investigation and security^25,26^.

Casino et al.^27^described In a comparative study, researchers evaluated the two approaches and concluded that the Hyper Ledger Blockchain Framework provided better scalability, an identity management system, better TPS (transactions per second), and accountability. This is because Hyper Ledger is designed for enterprise-level use and is better suited to handle large-scale supply chain transactions, while Ethereum is primarily designed for decentralized applications and has limitations in terms of scalability and identity management. Overall, the use of blockchain technology in the pharma supply chain is an exciting area of development, and the adoption of frameworks like HyperLedger can help to unlock the full potential of this technology in the pharma industry.

The paper, “Designing sustainable recovery network of end-of-life product during the COVID-19 pandemic: A real and applied case study,” authored by Abbasi, Sina, Maryam Daneshmand-Mehr, and Armin Ghane Kanafi, published in Discrete Dynamics in Nature and Society in 2022, presents a real-world case study on creating a sustainable recovery network for end-of-life products, focusing on the challenges posed by the COVID-19 pandemic. The research explores strategies and solutions for managing product recovery during these challenging times, offering insights into sustainable practices in the context of pandemic-induced disruptions. This study contributes to the discourse on supply chain resilience and sustainability during crisis situations^28^

Saini et al.^29^ proposed a The use of an Ethereum-based public blockchain for an organic food supply chain is an innovative application of blockchain technology. This solution enables stakeholders to track the movement of organic food products from the point of origin to the final destination and to ensure that the products are authentic and of high quality. The Solidity-based smart transaction model developed for this solution helps to validate products and ensure that they comply with industry standards and regulations. This adds an additional layer of security and accountability to the supply chain, and can help to prevent fraud and counterfeiting.

The paper by Shaikh, Zaffar Ahmed et al. discusses the challenges and limitations of implementing a blockchain Hyperledger-enabled e-healthcare application within the context of BIoMT (Blockchain-based Internet of Medical Things) modular infrastructure. It explores the complexities and issues associated with this technology in the healthcare sector. The study provides insights into the obstacles faced in developing a secure and efficient e-healthcare system using the Hyperledger blockchain. The authors highlight various challenges and limitations, shedding light on the intricacies of integrating blockchain into healthcare, ultimately contributing to the ongoing discourse on improving the healthcare industry through emerging technologies^30^.

Naz et al.^31^ proposed the use of an Ethereum-based permissioned blockchain to prevent counterfeit drugs in the pharmaceutical industry is a novel and promising application of blockchain technology. By leveraging the benefits of blockchain, this solution can help to improve the safety and efficacy of pharmaceutical products, and reduce the risk of counterfeit drugs entering the market. By using a permissioned blockchain, this solution can ensure that only authorized parties have access to sensitive information, and that transactions are validated and approved by trusted parties. This can help to prevent fraud and counterfeiting, and increase trust and confidence in the pharmaceutical supply chain.

Gaur et al.^32^, proposed usage of The use of IoT edge devices and blockchain to enhance traceability in the pharmaceutical supply chain is an innovative and promising solution to improve the transparency and accountability of the industry. By leveraging the benefits of IoT and blockchain, this solution can help to automate data collection, processing, and validation, and ensure the authenticity and integrity of data throughout the supply chain. Furthermore, by taking feedback from physicians and other beneficiaries, this solution can be continuously improved and refined to meet the needs of the industry. This can help to improve patient outcomes, reduce waste and inefficiencies, and increase the efficiency and productivity of the industry as a whole.

Composer^33^ put forward The systematic mapping study of the related parameters in the system architecture of the supply chain based on blockchain is an important research topic that can help to identify the key parameters and categories that are relevant to the development and implementation of blockchain-based solutions in the supply chain. By mapping the topics against research categories using a bubble plot, this study can provide valuable insights into the current state of research in this area. Interestingly, it was found that most of the articles were based on solutions such as Blockchain ledger and Ethereum. This suggests that these solutions are currently the most popular and widely used in the field, and that there is a need for further research and development in this area.

The research article titled “Performance Measurement of the Sustainable Supply Chain During the COVID-19 Pandemic: A real-life case study," authored by Abbasi, Sina, et al. and published in the "Foundations of Computing and Decision Sciences” in 2022, delves into the crucial evaluation of sustainable supply chain performance in the context of the COVID-19 pandemic. Through a real-life case study, the paper explores the impact of the pandemic on supply chains and assesses the effectiveness of sustainability measures. The study offers insights into how organizations coped with the disruptions caused by the pandemic, making it a valuable resource for understanding supply chain resilience and sustainability in challenging circumstances^34^.

Blossey et al.^35^ proposed a system to It analyzed various use cases of blockchain in supply chain management and identified issues related to data ownership and low data quality in supply chain settings. The focus was primarily on manufacturing and coordination within supply chain management.

The existing literature extensively covers the application of blockchain technology in supply chain management across various industries, such as pharmaceuticals, food, and manufacturing, there appears to be a noticeable research gap concerning the specific challenges related to data ownership and data quality within supply chain settings. Although these studies highlight the potential of blockchain to enhance transparency, traceability, and security in supply chains, they often lack in-depth analysis of how data ownership and data quality issues are addressed. Further investigation is needed to explore the mechanisms, protocols, and governance structures required to ensure secure and reliable data sharing and ownership rights in blockchain-based supply chain solutions. Addressing this research gap could significantly contribute to a more comprehensive understanding of the practical implementation of blockchain technology in supply chain management. So the proposed research evaluates the performance of the SCRM with blockchain across various test scenarios, featuring differing numbers of organizations and clients. Multiple fabric networks are employed to assess the system's scalability and performance under diverse conditions to map the objectives. The results of these comprehensive tests inform practical deployment decisions and highlight areas for potential optimization and further development. So this research work provides valuable insights into the application of blockchain in pharmaceutical supply chains, offering a roadmap for implementation and improving supply chain security, efficiency, and transparency.

## Materials and methods

### Pharmaceutical product supply chain logistics

The pharmaceutical product supply chain logistics refers to the processes involved in the movement and management of pharmaceutical products from their point of origin to the end-users, including patients, healthcare providers, and distributors. The supply chain logistics involve the planning, execution, and control of the movement of products, from the procurement of raw materials and production to the distribution and delivery of the final products. The pharmaceutical product supply chain logistics is critical to ensure the availability, safety, and efficacy of drugs. The pharmaceutical industry operates under strict regulatory requirements that aim to ensure the quality, safety, and efficacy of drugs. Therefore, the supply chain logistics must comply with these requirements to prevent the distribution of counterfeit or substandard drugs that can harm patients^27,28^.

**Figure 1:**
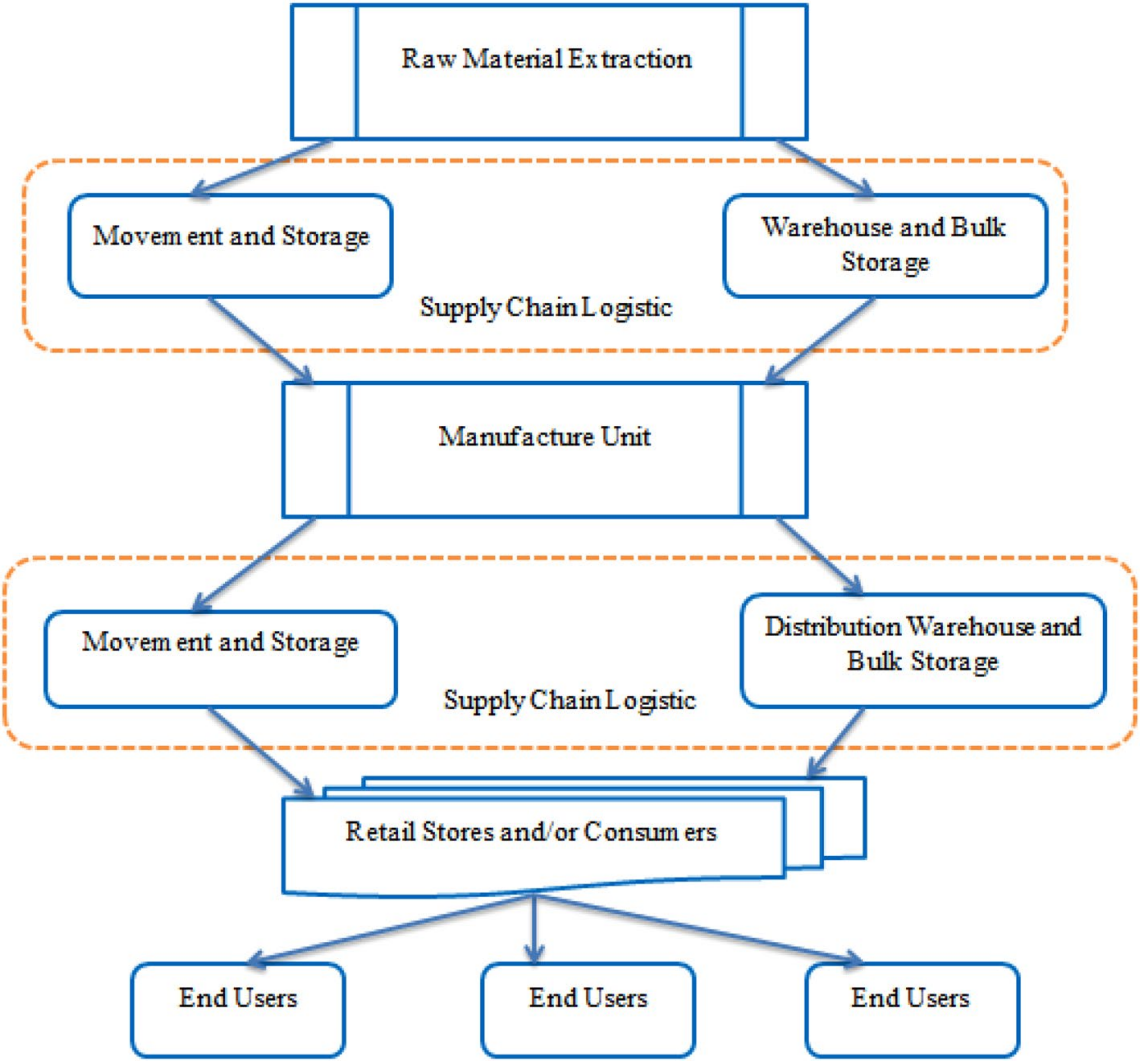
Product Supply Chain Logistics.

The supply chain logistics involves various stakeholders, including manufacturers, distributors, wholesalers, retailers, end users and transportation companies as shown in Fig. 1.

Each stakeholder has a role to play in ensuring the timely and efficient delivery of pharmaceutical products. For instance, manufacturers must ensure the quality and consistency of their products and comply with regulatory requirements. Distributors and wholesalers must manage inventory and ensure the availability of products. Transportation companies must ensure the safe and timely delivery of products. To improve the efficiency and effectiveness of pharmaceutical product supply chain logistics, companies are adopting various technologies, including track-and-trace systems, RFID, and blockchain. These technologies help to improve visibility, traceability, and accountability in the supply chain logistics, thereby reducing the risk of counterfeiting, diversion, and theft.

### Blockchain for supply chain

Blockchain technology can be used to restructure supply chain effective management by giving a transparent, secure and tamper-proof way to track and trace the movement of goods from the point of origin to the end-user^24^. The technology can improve supply chain efficiency, reduce costs, enhance transparency, and increase trust among the stakeholders. The following are some of the ways in which blockchain technology can be used in supply chain management:

#### Transparency and traceability

Blockchain technology can be used to create an immutable and transparent record of every transaction or event that occurs throughout the supply chain. This can help to enhance transparency, traceability, and accountability in the supply chain, thereby reducing the risk of fraud, errors, and disputes.

#### Improved efficiency

By using blockchain technology, supply chain stakeholders can automate many of the manual processes that are involved in managing the supply chain. This can help to improve supply chain efficiency, reduce costs, and enhance the speed of delivery.

#### Enhanced security

Blockchain technology uses advanced encryption algorithms to secure data and prevent unauthorized access. This can help to enhance the security of sensitive information such as product information, pricing, and transaction data.

#### Increased trust

Blockchain technology can help to increase trust among supply chain stakeholders by providing a secure and transparent platform for sharing information and conducting transactions. This can help to reduce the risk of disputes and increase collaboration among stakeholders.

#### Real-time visibility

By using blockchain technology, supply chain stakeholders can have real-time visibility into the movement of goods and inventory levels. This can help to improve inventory management, reduce the risk of stockouts, and enhance customer satisfaction.

So blockchain technology can provide a powerful tool for improving supply chain management. By enhancing transparency, traceability, efficiency, security, and trust, the technology can help to create a more resilient and reliable supply chain that can better serve the needs of businesses and customers alike.

## Blockchain for pharmaceutical product supply chain

The pharmaceutical product supply chain is a complex and highly regulated system that involves multiple stakeholders, including manufacturers, distributors, wholesalers, retailers, and end-users. The use of advanced technology like blockchain technology with security enhancement used to improve the efficiency and transparency in the pharmaceutical supply chain while ensuring compliance with regulatory requirements^7,29^. Here is a framework for using blockchain technology in the pharmaceutical product supply chain:

### Supply chain mapping

The first step in implementing a blockchain-based supply chain for pharmaceutical products is to map the supply chain. This involves identifying all the stakeholders involved in the supply chain and the flow of products and information between them. This information can be stored on a blockchain ledger, which provides a transparent and immutable record of the supply chain.

### Product identification and tracking

Blockchain technology can be used to track the movement of pharmaceutical products from the point of manufacture to the end-user. Each product can be given a unique identifier that is stored on the blockchain. This identifier can be used to track the product throughout the supply chain, enabling stakeholders to monitor the product's movement, storage conditions, and other critical information.

### Quality control and compliance

Blockchain technology can be used to ensure compliance with regulatory requirements and quality control standards. The blockchain can be used to store information about the quality control checks performed on the products and the regulatory certifications obtained by the stakeholders in the supply chain.

### Inventory management

The Blockchain for manage inventory is one of the important levels across the supply chain. Each stakeholder in the supply chain can access real-time inventory data, which can be used to optimize inventory levels and prevent stockouts.

### Smart contracts

It can be used to automate transactions and enforce business rules. For example, smart contracts can be used to automate the payment process between stakeholders in the supply chain or to enforce quality control standards.

### Data sharing and collaboration

Blockchain technology can be used to facilitate data sharing and collaboration between stakeholders in the supply chain. By providing a secure and transparent platform for sharing information, stakeholders can work together more effectively to manage the supply chain.

So by implementing a blockchain-based supply chain framework, pharmaceutical companies can improve the efficiency and transparency of the supply chain, enhance compliance with regulatory requirements, and ensure the quality and safety of pharmaceutical products.

## Shipping process of raw material in SCM

The shipping process involves various stakeholders, each playing a critical role in ensuring the timely and efficient movement of goods across borders. Here is a brief overview of the roles and responsibilities of each actor:

- **Importer:** The importer is responsible for purchasing goods from the exporter and arranging for their shipment.
- **Exporter:** The exporter is responsible for manufacturing or sourcing goods and arranging for their shipment to the importer.
- **Bank:** The bank provides financing and payment services for the importer and exporter, including issuing letters of credit and processing payments.
- **Insurance Company:** The insurance company provides insurance coverage for the goods during transit, protecting against loss or damage.
- **Freight Forwarder:** The freight forwarder is responsible for arranging and coordinating the shipment of goods, including booking cargo space, preparing documentation, and arranging for transportation.
- **Shipping Company:** The shipping company provides the actual transportation of the goods by sea or air, including loading and unloading of cargo.
- **Customs House Agent (CHA):** The CHA is responsible for clearing the goods through customs and ensuring compliance with customs regulations.
- **Customs Authorities:** The customs authorities are responsible for enforcing customs regulations and collecting duties and taxes on imported goods.
- **Port Authorities:** The port authorities are responsible for managing and maintaining the port facilities and infrastructure, including cargo handling equipment and berths.
- **Intermodal Transport Providers:** Intermodal transport providers provide transportation services for goods using multiple modes of transport, such as sea, air, rail, and road.

**Figure 2:**
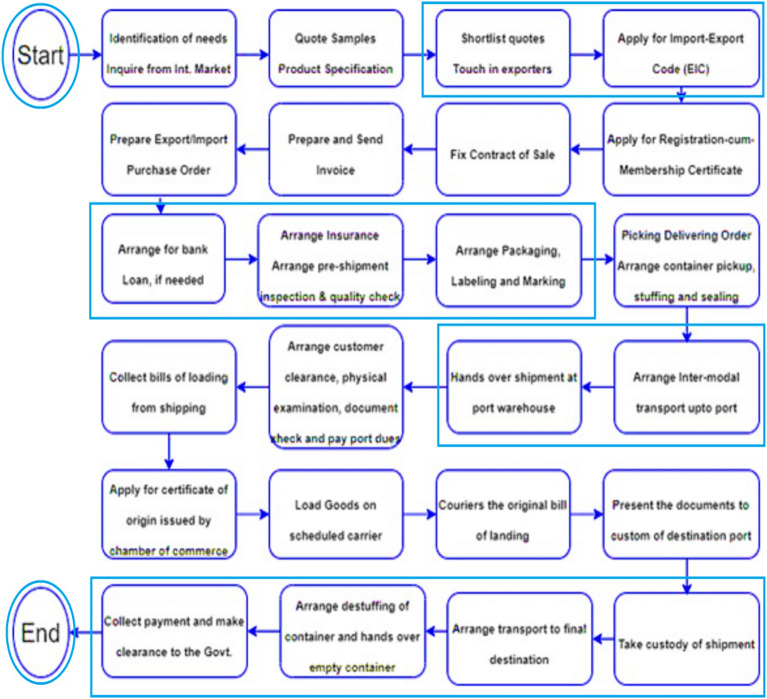
The Shipping Process of Supply Chain Logistics.

Each actor in the shipping process plays a critical role in ensuring the efficient movement of goods across borders. By working together effectively, they can help to minimize delays, reduce costs, and ensure the timely delivery of goods to their destination. A detailed process is shown in Fig. 2

Maintaining a secure chain of information exchange is critical in ensuring the integrity and safety of the raw materials being transported. As you mentioned, the use of a blockchain-based supply chain risk management (SCRM) system can help to achieve this goal by providing an immutable and transparent record of all information exchanges. By using a blockchain-based SCRM system, all information exchanges related to the raw materials can be recorded in a tamper-proof and secure manner, with each exchange forming a block in the blockchain. This ensures that the information remains transparent and accessible to all relevant actors while also ensuring that the integrity of the data is maintained. Moreover, because the blockchain is decentralized, there is no single point of failure or potential for a single actor to manipulate the data^26,27,36,37^. Which adds an extra security layer to the information exchange process, reducing the risk of fraudulent activity or data tampering? Overall, the use of a blockchain-based SCRM system can provide a highly secure and transparent chain of information exchange for raw materials, helping to ensure the integrity and safety of the products being transported.

**Figure 3:**
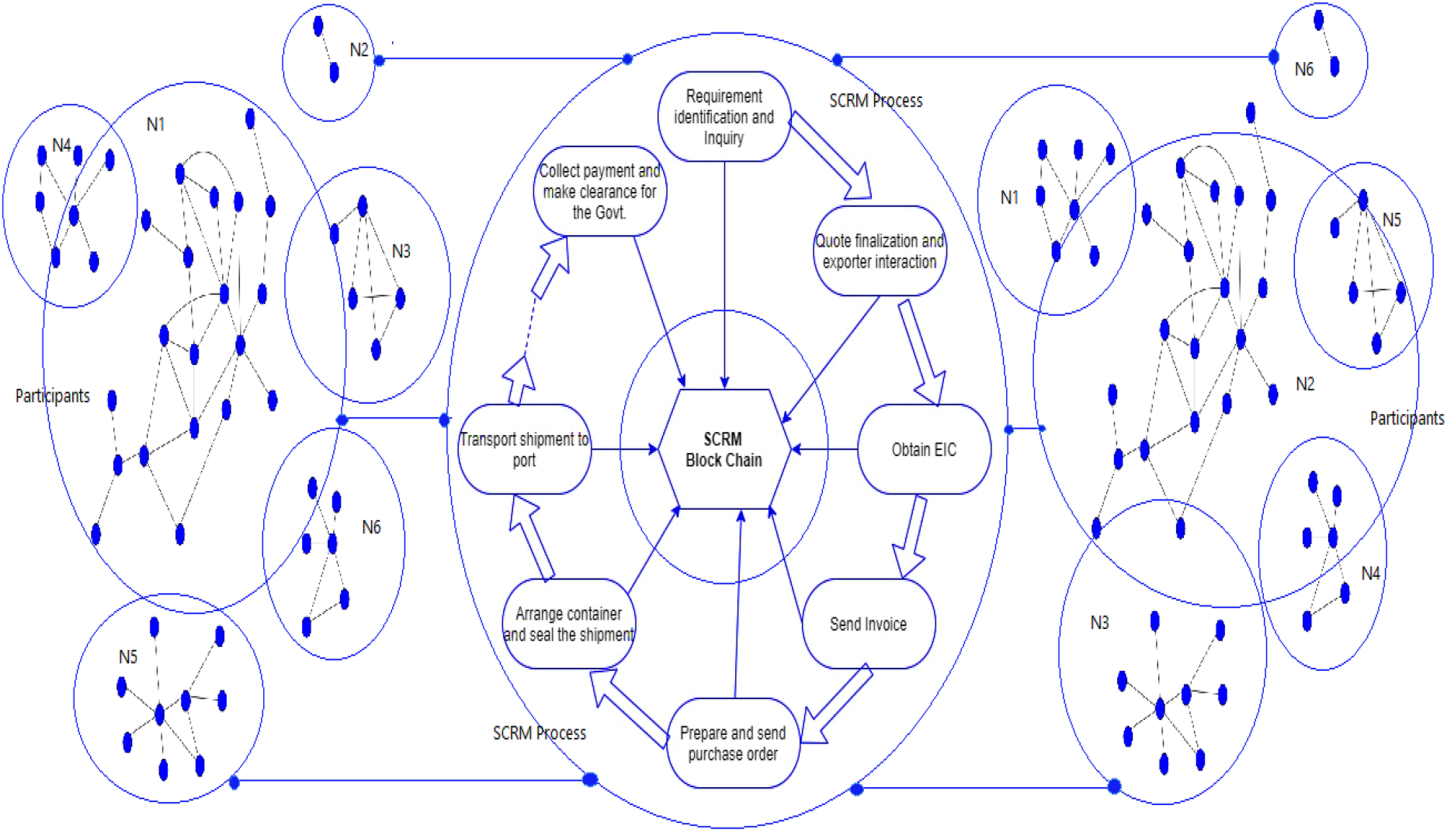
Conceptual Model for SCRM block chain for raw material shipment.

Figure 3 outlines the detailed process of raw material shipping and how it can be used to track pharmaceutical products in the supply chain, from the origin to destination, while involving all the stakeholders from different organizations involved in the systematic process^29^. Also, the shipping process is explained in the form of SCRM model, where record keeping of all the stages including from selecting the raw material to reporting to different stakeholders is available in the diagram.

### Hyperledger composer

**Figure 4:**
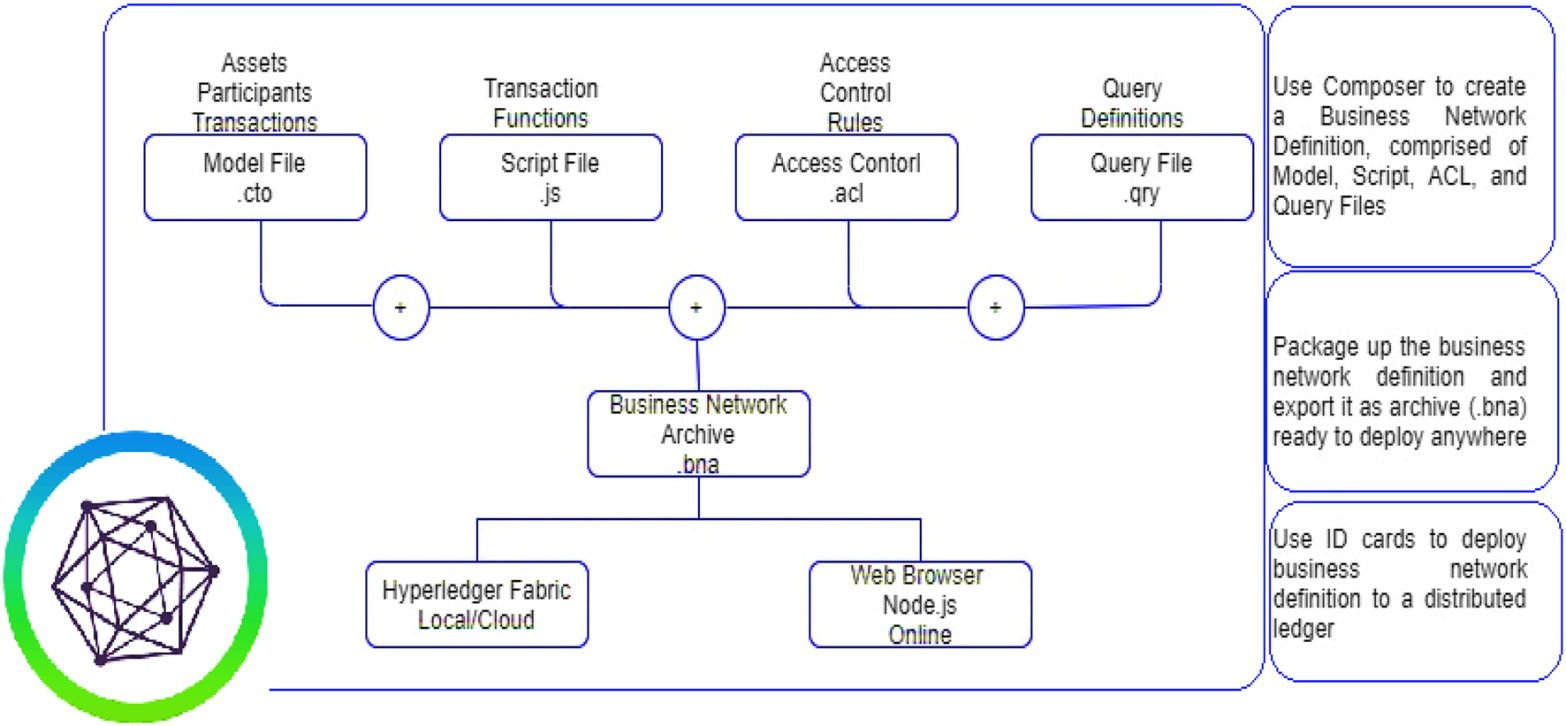
Framework of Hyperledger Composer.

Hyperledger Composer is a powerful open-source toolset that is specifically designed for developing blockchain applications. It provides developers with a range of tools and libraries to help them create and deploy blockchain applications rapidly. One of the key features of Hyperledger Composer is its ability to integrate data with blockchain applications. This is achieved by creating business networks that contain assets, stakeholders, and communications across multiple blockchain networks. These networks are then recorded in the Hyperledger Composer, which provides a centralized repository for all blockchain-related data^29,31^. Hyperledger Composer also provides a range of tools for managing transactions between different stakeholders, such as sellers, buyers and observers/viewers in the blockchain network. Once these parties have been registered in the blockchain, they can carry out transactions as per their roles and responsibilities. The Hyperledger Composer framework works in three layers, as mentioned in Fig. 4. The composer in the first layer is used to create a business-network definition, which includes the model, script, ACL (Access Control List), and query files. The model file defines the data model used in the application, the script file defines the transaction logic, the ACL file defines the permissions and access control rules, and the query file defines the queries that can be used to retrieve data from the ledger. The second layer it is packaged up into an archive file format that can be exported and deployed to any environment that supports the Hyperledger Fabric blockchain network^29,38^. The final layer uses ID cards to deploy the business network definition to a distributed ledger. An ID card is essentially a digital identity that is used to authenticate and authorize users and applications to interact with the blockchain network. Once the ID card is created, it can be used to deploy the business network definition to the blockchain network. Overall, Hyperledger Composer provides a powerful and flexible framework for developing blockchain applications, with a range of tools and libraries to help developers create, manage, and deploy blockchain networks quickly and efficiently.

### Tool for performance evaluation of blockchain

Several tools available for the performance evaluation of blockchain, some of which are:

- **Hyperledger caliper—**Hyperledger Caliper is an open-source blockchain performance benchmark tool that allows developers to measure and analyze the performance of different blockchain solutions. It supports various blockchain platforms, including Hyperledger Fabric, Ethereum, and others.
- **Blockchain bench—**Blockchain Bench is another open-source benchmark tool that enables developers to test and evaluate the performance of blockchain systems. It allows users to customize the test parameters and simulate various scenarios to identify the strengths and weaknesses of different blockchain solutions.
- **Blockbench**—Blockbench is a benchmark suite for private and consortium blockchains, which provides a comprehensive set of benchmarks for measuring the performance of different blockchain platforms. It supports Hyperledger Fabric, Ethereum, and other platforms.
- **Chainhammer**—Chainhammer is an open-source blockchain benchmark tool that is designed to test the performance and scalability of Ethereum-based blockchain networks. It uses smart contract transactions to simulate realistic workloads and evaluate the performance of the network.
- **TPC-BC**—TPC-BC is a standard benchmark tool for evaluating the performance of blockchain systems, developed by the Transaction Processing Performance Council (TPC). It provides a standard workload and metrics for measuring the performance of blockchain systems.

Overall, these tools provide a range of features and functionalities for testing and evaluating the performance of blockchain systems, enabling developers to optimize their blockchain solutions and ensure that they meet the required performance standards. Performance of the blockchain technology is an important challenge and a concern before deployment and implement anywhere it needs a tool. In our assessment, we leveraged Hyperledger Caliper to gauge the performance of the proposed blockchain model, which adheres to a predefined data structure. Hyperledger Caliper offers various reports that encompass a range of performance metrics, such as time per transaction (TPS), transaction latency, and resource utilization. These reports serve as valuable criteria for determining the appropriateness of the blockchain platform in accordance with the user's specific requirements.

### Evaluating the viability of blockchain technology for shipping raw material

A feasibility test of blockchain for shipping raw materials would typically involve an assessment of the potential benefits and drawbacks of implementing a blockchain-based system for managing the shipping process^22^. The following are some factors that could be considered in such a feasibility test:

- **Transparency:** One of the key benefits of blockchain is that it provides a transparent and immutable record of transactions. In the case of shipping raw materials, this could help to increase transparency throughout the supply chain and reduce the risk of fraud or theft.
- **Traceability:** Blockchain could also be used to track the movement of raw materials through the supply chain, from the point of origin to the final destination. This could help to improve traceability and accountability, as well as reduce the risk of counterfeit or substandard products entering the market.
- **Efficiency:** Blockchain could potentially improve the efficiency of the shipping process by reducing the need for intermediaries and streamlining documentation and communication between different stakeholders. This could lead to faster and more cost-effective shipping of raw materials.
- **Security:** Blockchain is a secure technology that uses advanced cryptography to protect against hacking and data tampering. This could provide an added layer of security to the shipping process and help to reduce the risk of cyber-attacks.
- **Adoption:** One of the challenges of implementing a blockchain-based system for shipping raw materials is that it requires widespread adoption across the supply chain. This may require significant investment and collaboration between different stakeholders to ensure that the system is effective and efficient^39^.

Overall, a feasibility test of blockchain for shipping raw materials for pharmaceutical supply chain logistics would need to consider these factors and assess the potential benefits and drawbacks of implementing a blockchain-based system for managing the shipping process. This would involve evaluating the costs, benefits, and risks of such a system and determining whether it is feasible and cost-effective in the long run.

## Proposed model

**Figure 5:**
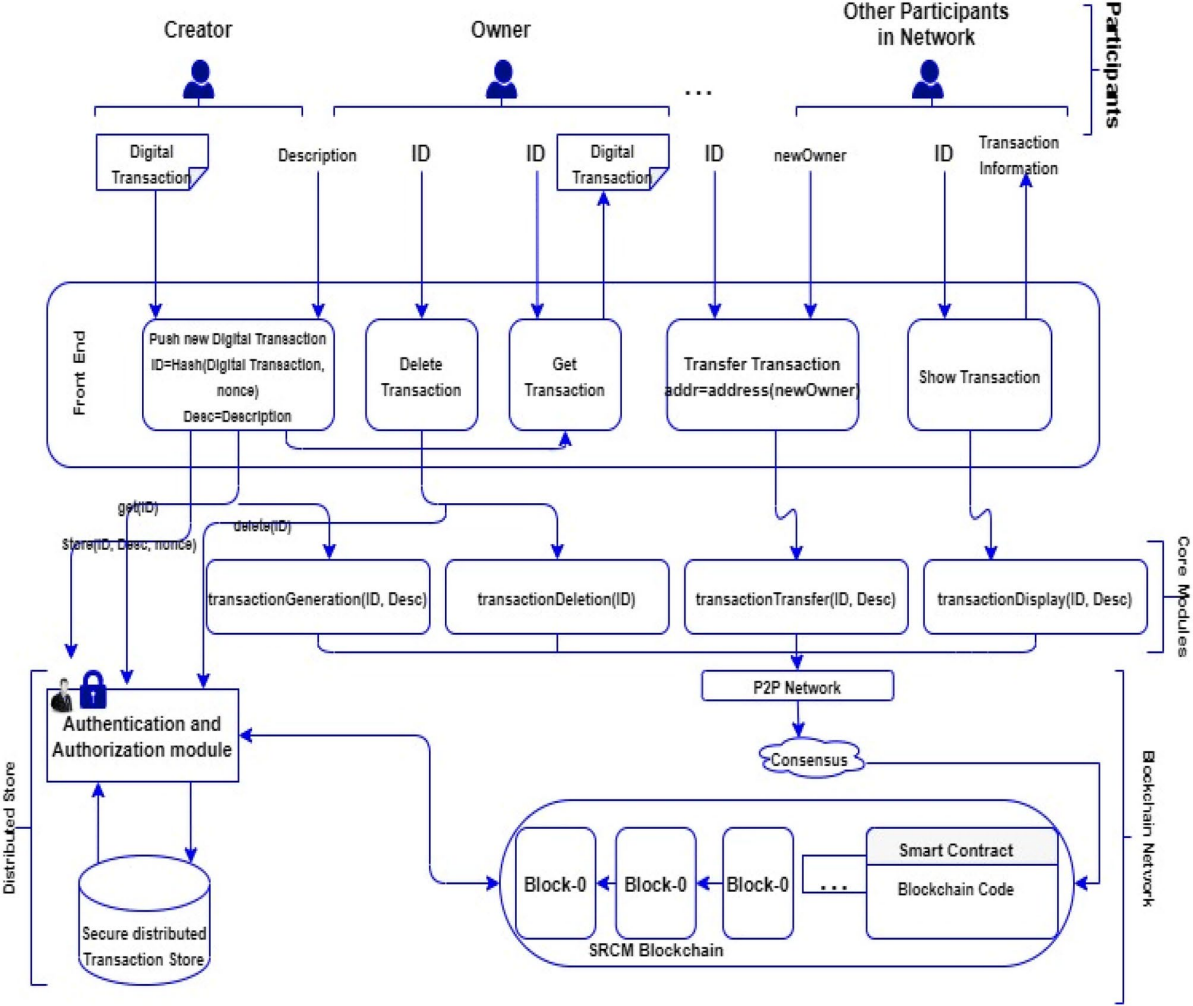
Functional Flow diagram of SCRM Blockchain Technology.

The proposed model comprised of assets, participants, and transactions, all stored within the blockchain. The SCRM Blockchain Technology's functional flow diagram includes Core Modules, Blockchain Module, Distributed Transaction Store, and Front End^23^. Core Modules manage functionalities, Blockchain Module handles blockchain operations, Distributed Transaction Store stores data securely, and Front End provides user interaction. Together, these components enable secure and transparent handling of assets, participants, and transactions in blockchain applications as shown in Fig. 5.

**Figure 6:**
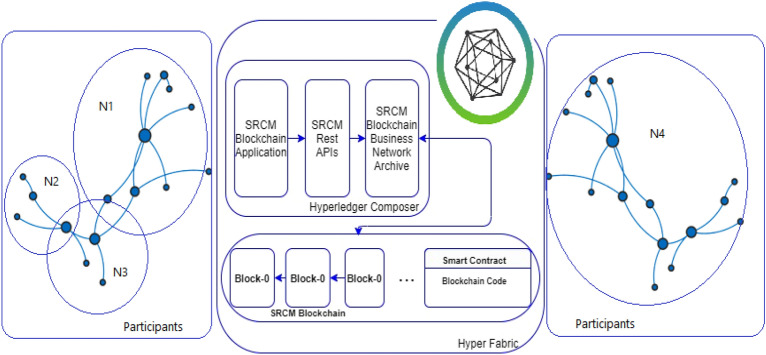
The Communication of SCRM blockchain model with stakeholders.

- **Core Modules:** These modules in SCRM are required for monitoring the communication and coordination among the nodes or participants in the network. These modules are typically designed to handle key functions such as transaction validation, block creation, and consensus mechanisms. Functional Flow diagram of SCRM Blockchain Technology is shown in Fig. 6
- **Blockchain Module:** This module is used for creating new blocks in the blockchain. It collects valid transactions from the transaction processing module and adds them to a new block.
- **Distributed Transaction Store:** This module is responsible for validating and processing transactions submitted by participants. It ensures that each transaction is valid and follows the rules of the blockchain network.
- **Front End:** This interface allows the participants to perform various actions on the SCRM blockchain, such as creating new assets or participants, updating or deleting existing ones, and submitting transactions. The front end also provides real-time visibility into the state of the blockchain, allowing participants to monitor the progress of their transactions and view the current state of the system. Overall, the front end plays a crucial role in enabling seamless interaction between the participants and the SCRM blockchain. Figure 6 shows the Communication of participants with SCRM blockchain UI.

### Proof-of-concepts

The SCRM (Supply Chain Risk Management) blockchain application can be implemented as a proof-of-concept (PoC) to demonstrate its feasibility and potential benefits. The PoC can involve a small-scale simulation of the supply chain process, using mock data and participants. The following steps can be taken to implement the PoC:

- Define the scope and objectives of the PoC, such as the specific supply chain process to be simulated, the expected benefits of using blockchain, and the key performance indicators (KPIs) to measure success.
- Design the blockchain architecture and smart contract code, using tools such as Hyperledger Composer or Solidity. Define the data structure for assets, participants, and transactions, and ensure that the smart contract enforces the desired business rules and conditions.
- Implement the blockchain network and deploy the smart contract code on it. Set up the necessary nodes, such as orderers and peers, and configure the permissions and access controls.
- Create the mock data and participants, including assets, such as raw materials, products, and documents, and participants, such as suppliers, manufacturers, and distributors. Define their identities and roles in the supply chain process.
- Conduct the simulation, using the mock data and participants, and record the transactions on the blockchain. Monitor the performance and accuracy of the system, and collect data on the KPIs.
- Analyze the results and evaluate the success of the PoC. Assess the performance and scalability of the system, as well as the benefits and challenges of using blockchain for SCRM. Identify any improvements or modifications that may be needed for future implementation.
- Present the PoC findings and recommendations to stakeholders, such as supply chain managers, regulators, and investors, and seek their feedback and support for further development and implementation.

By conducting a PoC for SCRM using blockchain, organizations can gain valuable insights into the potential benefits and challenges of using this technology for supply chain management. This can help them make informed decisions about whether to invest in blockchain, and how to design and deploy it effectively. The data structure is used to define a SCRM transaction model as shown below.

### Description of terminology used

The terminologies in the process involved are mentioned and defined in the Table 1^22^

**Table 1:** Terminology used data structure.

Terminology	Description
#TransactionID	Transaction ID (or transaction hash) is a unique identifier generated by applying a cryptographic hash function to the transaction data. However, the specific hash function used to generate the transaction ID may not necessarily be SHA256, as different blockchain networks may use different cryptographic hash functions
#Creator	The creator of a transaction is typically the participant who initiates the transaction by sending a request to the network to perform a specific action, such as transferring funds or executing a smart contract
#Owner	It is the stakeholders who possess the transaction presently
#Transaction Description	The stakeholder includes the necessary requirements to define a transaction
#Case ID	Case ID is a unique number assigned to a raw material shipment. It is used to initialize the smart contract
#Transfer Chain	Transfer Chain includes the address of the stakeholders for the transaction and it can be stored in an array
#Transfer Time	Transfer Time records the date and time of for transaction and it can be stored in an array

The SCRM model is implemented using the Hyperledger Composer blockchain development tool, which provides a user-friendly and intuitive interface for creating and deploying blockchain-based applications^40,41^. The model has essential functionalities including creating, updating, deleting, and displaying transactions from the blockchain, and access to these functionalities is controlled by the permissions.acl file. Different class structures and script segments are used to define the data model and transaction logic for the SCRM blockchain are shown below.

### Sample codes


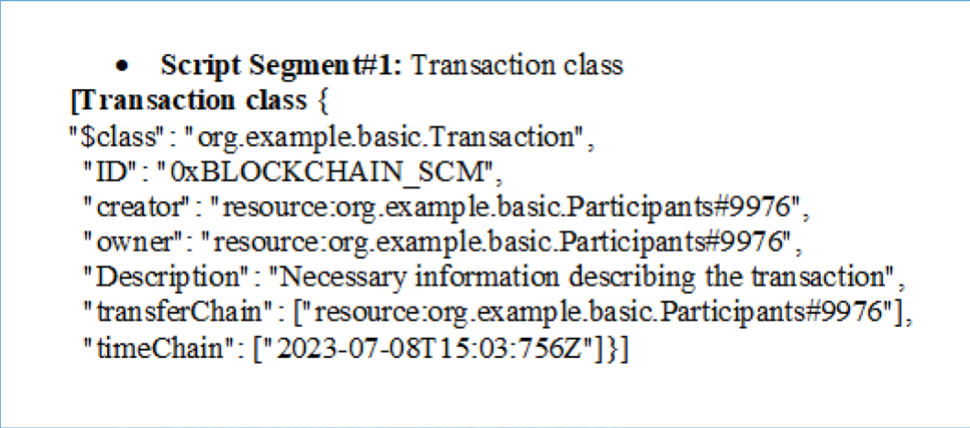


The provided script segment describes a transaction represented in a class format. Here's the breakdown of its key components:

- **$class:** This field specifies the class type of the transaction, which is "org.example.basic.Transaction." It indicates the type or category of the transaction within the system or application.
- **ID:** The "ID" field contains a unique identifier for the transaction, set to "0xBLOCKCHAIN_SCM." This identifier can be used to distinguish this specific transaction from others within the system.
- **creator:** This field identifies the creator of the transaction. It is set to "resource:org.example.basic.Participants#9976," indicating that the participant with the ID "9976" is the one who initiated or created the transaction.
- **owner:** The "owner" field specifies the owner of the transaction. In this case, it is also set to "resource:org.example.basic.Participants#9976," indicating that the same participant (ID: 9976) currently owns the transaction.
- **Description:** This field provides a brief description or necessary information about the transaction. The description is "Necessary information describing the transaction," which can include details about the purpose, content, or nature of the transaction.
- **transferChain:** The "transferChain" field is an array that lists participants involved in the transaction. In this example, it contains a single entry, "resource:org.example.basic.Participants#9976," indicating that the participant with ID 9976 is part of this transaction.
- **timeChain:** The "timeChain" field is another array that records timestamps related to the transaction. It includes a single timestamp, "2023–07-08T15:03:756Z," which represents the date and time when the transaction occurred.

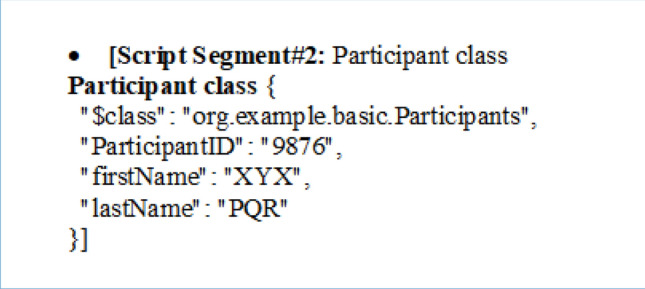


The provided script segment represents a participant class in a blockchain data structure. Here's the meaning of the fields in this class:

- **"$class":** This field specifies the class or type of the asset, which is "org.example.basic.Participants" in this case, indicating the class to which this participant belongs.
- **"ParticipantID":** This is a unique identifier for the participant, which is set to "9876" in this instance. It is used to uniquely identify this participant within the blockchain network.
- **"firstName":** This field holds the first name of the participant, which is set to "XYX" in this example.
- **"lastName":** This field contains the last name of the participant, which is set to "PQR" in this case.

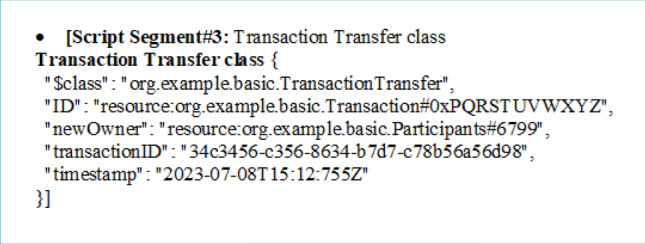


The provided script segment appears to describe a "Transaction Transfer" class in a blockchain-based system. Here's an explanation of the key elements:

- **$class:** This field specifies the class of the transaction, indicating that it belongs to the "org.example.basic.TransactionTransfer" class.
- **ID:** This field represents the unique identifier for the transaction transfer and is linked to a specific transaction. It uses a resource reference to point to a particular transaction, identified by its ID, which is "0xPQRSTUVWXYZ" in this case.
- **newOwner:** It identifies the new owner of the transaction. This field uses a resource reference to link to a participant or user in the system, identified as "org.example.basic.Participants#6799."
- **transactionID:** This field contains a unique identifier for the transaction transfer itself. The identifier "34c3456-c356-8634-b7d7-c78b56a56d98" is used to distinguish this specific transfer from others.
- **Timestamp:** This field indicates the date and time when the transaction transfer occurred. In this case, it occurred on "2023–07-08" at "15:12:755" (in UTC time zone).

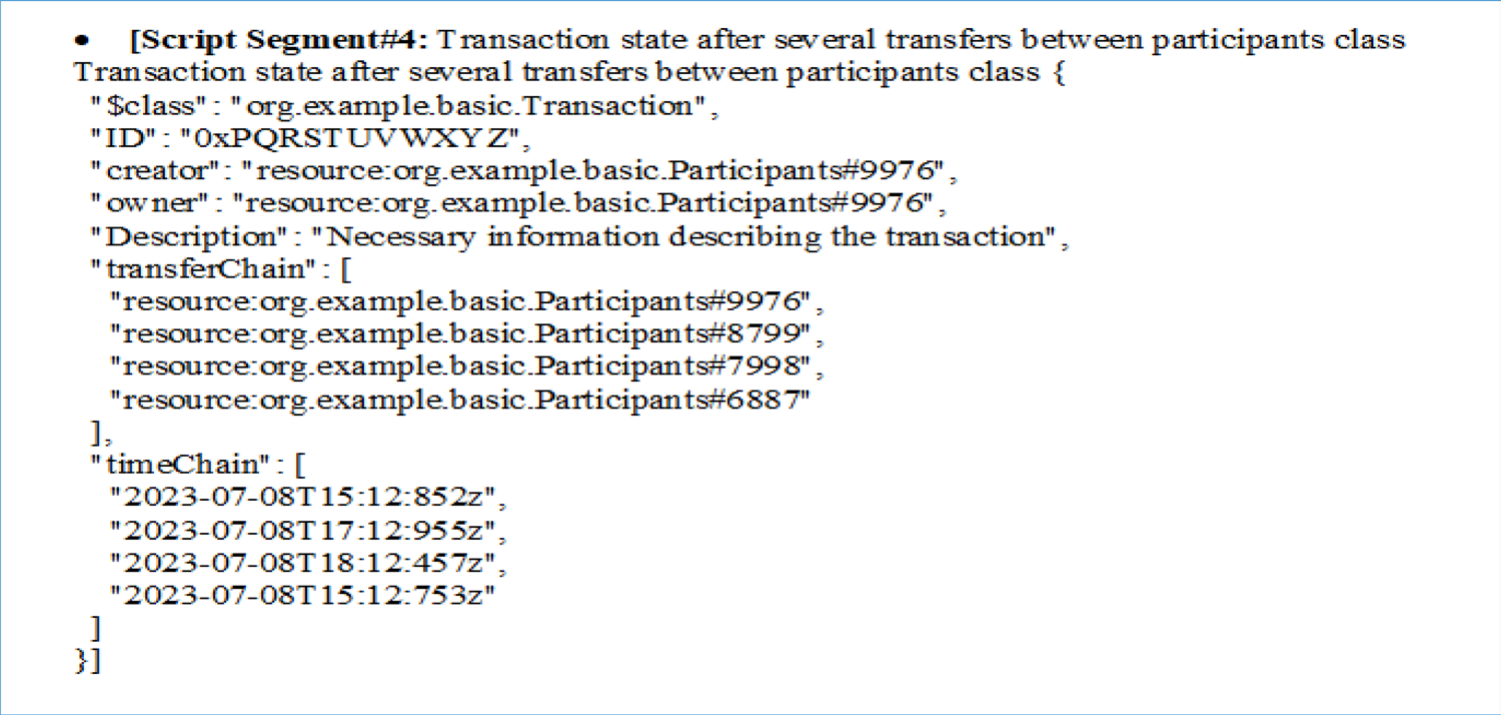


The provided script segment describes the state of a "Transaction" after several transfers between participants in a blockchain-based system. Here's an explanation of the key elements:

- **$class:** This field specifies the class of the transaction, indicating that it belongs to the "org.example.basic.Transaction" class.
- **ID:** This field represents a unique identifier for the transaction. In this case, the ID is "0xPQRSTUVWXYZ."
- **creator:** It indicates the participant who initially created the transaction. The "creator" is linked to a specific participant identified as "org.example.basic.Participants#9976."
- **owner:** It represents the current owner of the transaction. The "owner" is also linked to a specific participant, again identified as "org.example.basic.Participants#9976." It appears that the owner hasn't changed in this instance.
- **Description:** This field contains a description or necessary information describing the transaction, providing context or details about the transaction's purpose or content.
- **transferChain:** This is an array that represents a chain of participants involved in transferring ownership of the transaction. It shows the historical sequence of participants who have had ownership of the transaction. The array includes references to participants such as "org.example.basic.Participants#9976," "org.example.basic.Participants#8799," "org.example.basic.Participants#7998," and "org.example.basic.Participants#6887."
- **timeChain:** This array corresponds to the timestamps for each transfer of ownership within the "transferChain." It indicates when each transfer occurred. The timestamps are provided in the "2023–07-08" date format, along with the time in UTC, for example, "15:12:852z."

So this script segment represents the state of a blockchain transaction, tracking its history of ownership transfers among participants, along with associated timestamps, and provides a description of the transaction. It offers a transparent and immutable record of the transaction's lifecycle within the blockchain system.

**Figure 7:**
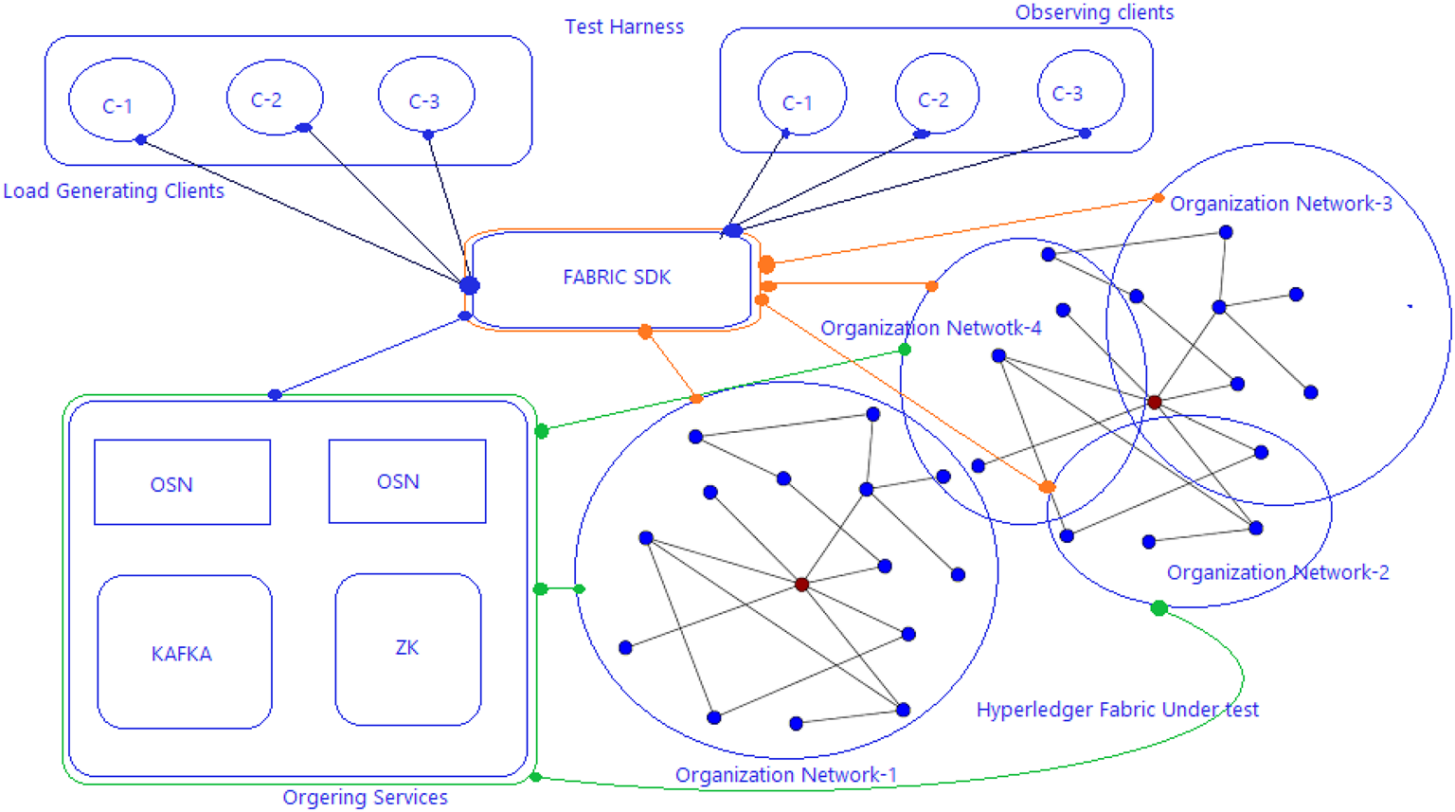
Hyperledger Framework for Fabric VI performance evaluation^14^.

This consortium governs the controlled environment where a permissioned SCRM blockchain is constructed using Hyperledger Composer. Framework for Hyperledger Fabric VI performance evaluation is shown in Fig. 7.

### Performance evaluation

Due to the unique properties of blockchain technology, traditional performance metrics may not be directly applicable. In a blockchain network, there is no central authority to validate transactions, and instead, each transaction is replicated across multiple nodes belonging to different organizations. Instead of processing individual transactions, blockchain networks typically process blocks of transactions that are committed after a consensus process involving all nodes on the network^18,19^. This consensus process ensures that all nodes on the network agree on the current state of the blockchain, which helps to maintain the integrity and security of the system. the consensus process is dependent on factors such as the input traffic and the hardware performance of the blockchain miner, it can be difficult to predict the exact time it will take for a transaction to be committed. However, by monitoring performance metrics such as the number of transactions in the queue, the block size, and the overall throughput of the network, it is possible to gain insight into the performance and scalability of the blockchain^20,22^.

### Experimental setup

The REST interface is used to interact with the blockchain network through HTTP requests, which enables easy integration of client applications with the blockchain network. The test harness is used to simulate the real-world scenarios and measure the performance of SCRM blockchain under different workloads and network conditions. The Hyperledger Fabric VI blockchain is used because it provides a high-performance, scalable, and secure platform for developing and deploying blockchain applications. It is an open-source, permissioned blockchain platform that allows organizations to build and deploy their own blockchain networks with fine-grained access control and confidentiality. Performance parameter of the blockchain is given as below^22^.

### Transaction latency

Transaction latency is the time it takes for a blockchain network to commit a transaction after it has been submitted. In other words, it is the time period between when a transaction is submitted to the blockchain network and when it is successfully added to the blockchain ledger. Average transaction latency in a blockchain network are committed in blocks after reaching consensus, it can be challenging to determine the transaction latency for individual transactions. Therefore, the average transaction latency is calculated by taking the sum of all transaction latencies and dividing it by the total number of committed transactions. This provides an estimate of the average time it takes for transactions to be added to the blockchain ledger and it is defined as below

$$\left[ {Average\,Transaction\,Latency \, = \, \left( {\sum Transaction\,Latency} \right)/Total\,committed\,Transactions} \right]$$

### Transaction throughput

Transaction throughput is the rate at which valid transactions are committed to the blockchain network over a defined time interval. It represents the number of transactions that the blockchain network can process in a given period of time^22^ and it is give as below-

$$\left[ {Transaction\,throughput \, = \, Total\,Committed\,Transactions \, / \, Total\,Time\,@\,percentage\,of\,committed\,transactions} \right]$$

### Scalability

Scalability is a measure of how well a blockchain system can maintain low transaction latency even with an increased number of workloads. This is an important consideration for blockchain systems, as increased workloads can lead to increased transaction processing times and lower system performance. By designing systems that can scale to accommodate higher workloads, blockchain developers can ensure that their systems remain performant and responsive even as demand grows^23^.

**Figure 8:**
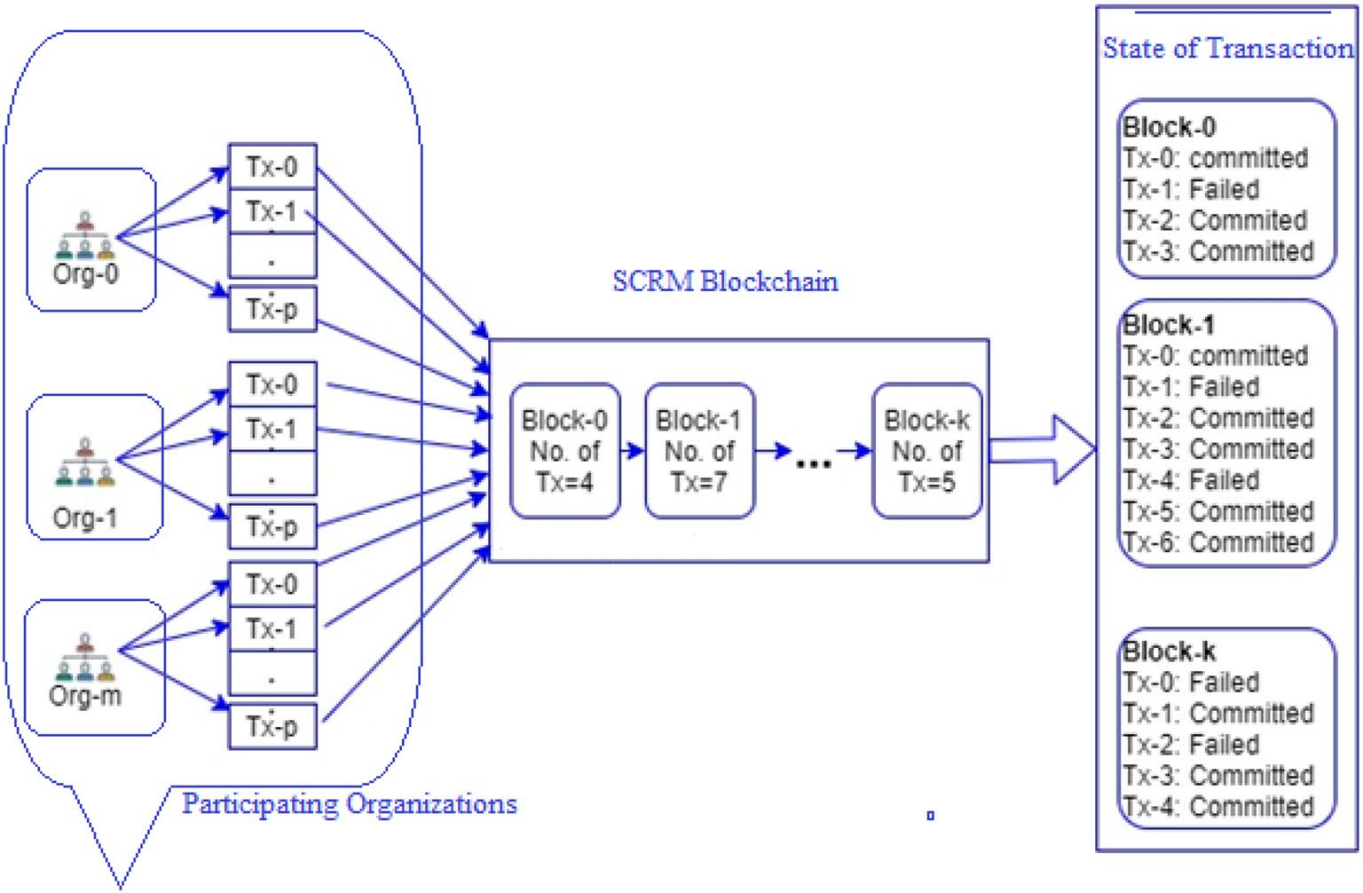
Pictorial Representation of Performance Metric.

In the pharmaceutical supply chain logistics, many organizations may be involved in transporting raw materials, and multiple participants from these organizations may submit transactions to the SCRM blockchain. These transactions then undergo a consensus process to ensure that they are valid and correct. Once the consensus process is completed, the transactions are assigned a state, which can either be committed or failed is depicted in Fig. 8.

The research conducted to test the performance of SCRM used four different scenarios, each with varying numbers of organizations and clients, and a total of 10 test runs for each scenario. The tests were conducted on a system running Ubuntu 16.04, with an Intel Core i5-7400 CPU, 8 GB of RAM, and multiple fabric networks were used to measure the performance of the SCRM blockchain. By conducting these tests on multiple fabric networks and with varying numbers of organizations and clients, the researchers were able to assess the performance and scalability of the SCRM blockchain under a range of different conditions. This information can be used to inform decisions about the deployment of SCRM in real-world settings, and to identify areas where further optimization or development may be necessary^22^. Tables 2 and 3 represents the different network involved and the values achieved for average latency and throughput for the all four scenarios.

**Table 2:** Types of Network involved.

Network type	Organizations (participate)	Clients involved	Block size	Test runs
Network-1	1	2	5	10
Network-2	2	6	5	10
Network-3	3	10	5	10
Network-4	4	24	5	10
Network-5	1	2	10	10
Network-6	2	6	10	10
Network-7	3	10	10	10
Network-8	4	24	10	10
Network-9	1	2	15	10
Network-10	2	6	15	10
Network-11	3	10	15	10
Network-12	4	24	15	10
Network-13	1	2	20	10
Network-14	2	6	20	10
Network-15	3	10	20	10
Network-16	4	24	20	10
Network-17	1	2	25	10
Network-18	2	6	25	10
Network-19	3	10	25	10
Network-20	4	24	25	10

**Table 3:** Values achieved for average latency and throughput for the all four scenarios.

Network Name	Block size	Tx(tps)	Tx time	Avg Latency	Throughput
Network-1	5	8	40	1.8	4
Network-2	5	48	226	9.81	9
Network-3	5	176	365	35.68	8
Network-4	5	257	687	51.77	6
Network-5	10	3	33	0.39	7
Network-6	10	38	228	3.97	13
Network-7	10	187	379	19.19	17
Network-8	10	289	702	29.31	23
Network-9	15	9	58	0.76	28
Network-10	15	43	287	3.02	32
Network-11	15	165	366	11.45	4
Network-12	15	317	764	21.55	7
Network-13	20	7	54	0.48	13
Network-14	20	46	276	2.47	16
Network-15	20	199	403	10.44	24
Network-16	20	297	701	15.27	12
Network-17	25	11	65	0.61	18
Network-18	25	54	305	2.34	26
Network-19	25	178	376	7.59	36
Network-20	25	307	735	12.7	42

**Figure 9:**
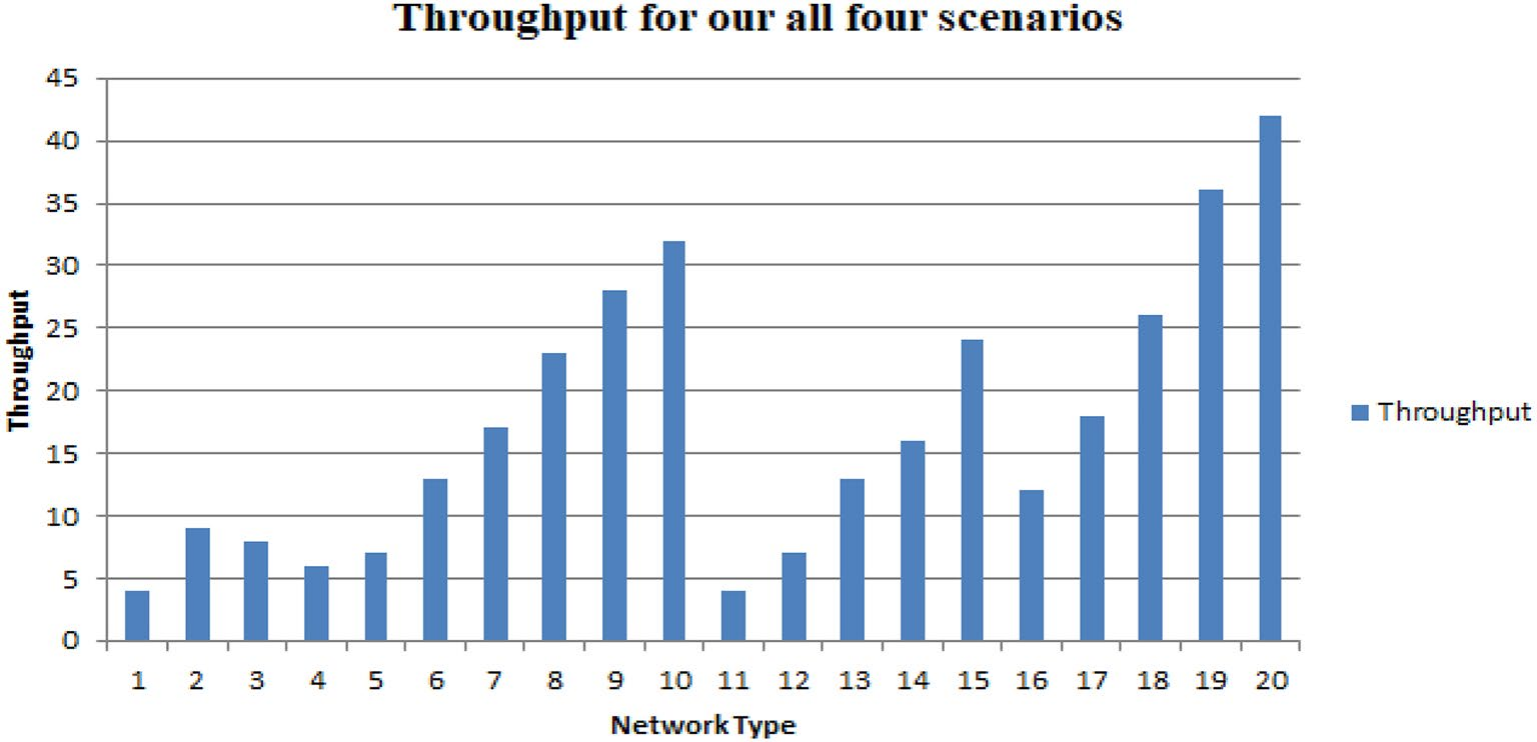
Throughput for our all four scenarios.

It appears that the nature of the SCRM is undulate with respect to throughput, as increasing the block size results in an increase in throughput for the same type of network across different scenarios. The used pattern seems to hold true for different types of networks as well. Therefore, it appears that the SCRM is able to maintain consistent performance with respect to throughput regardless of the network conditions as depicted by Fig. 9.

**Figure 10:**
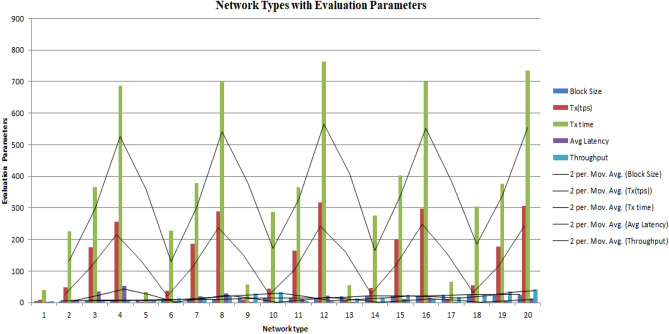
Scaling effects for different block sizes.

As depicted in Fig. 10 it shows that for block sizes ranging from 5 to 25, the SCRM is able to support scalability effectively. Specifically, for a block size of 5, 1.8 ms is the minimum average latency and 51.77 ms is the maximum average latency. Additionally, the throughput range is from 4 to 9 transactions for all four scenarios. Similarly, similar patterns are observed for other block sizes ranging from 10 to 25. These results suggest that the SCRM is able to maintain consistent performance and scalability across a range of block sizes^22^

**Figure 11:**
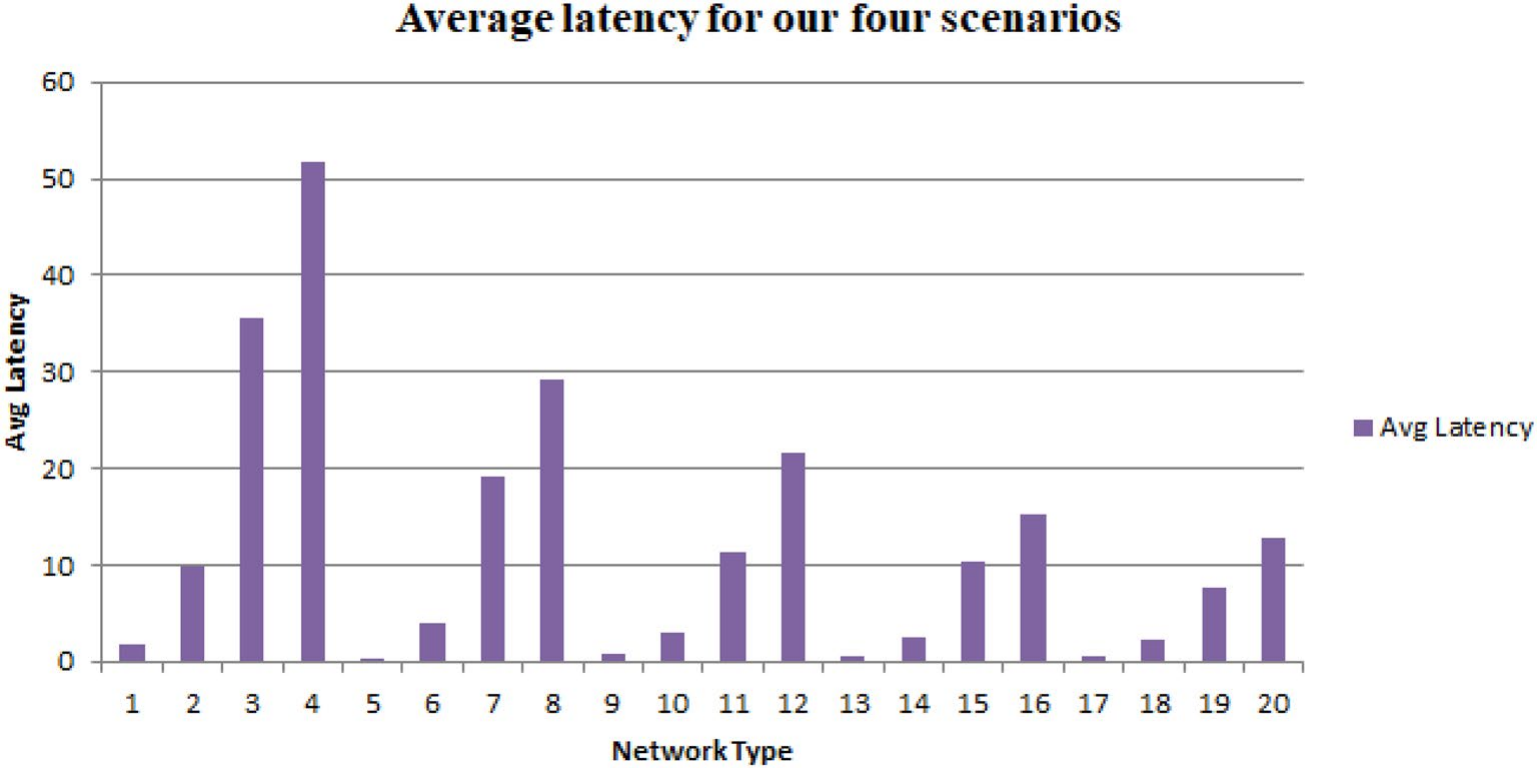
Average latency for our four scenarios.

The nature of the SCRM is undulate with respect to average latency, regardless of the type of network being used. Specifically, increasing the block size results in an increase in average latency across all network types. However, the behaviour of the SCRM remains consistent and does not fluctuate. This suggests that the SCRM is robust and reliable, as it is able to maintain consistent performance despite changes in network conditions and this is depicted by Fig. 11.

The proposed Supply Chain Risk Management (SCRM) solution using blockchain technology offers significant advantages over traditional solutions like relational databases, particularly in the context of pharmaceutical product supply chains. Blockchain's transparency, security, and scalability aspects make it a compelling choice for enhancing the integrity and efficiency of supply chain logistics in the pharmaceutical industry. Blockchain's transparency is a key advantage. In a relational database system, transaction details are typically not visible to all users, leading to potential discrepancies and lack of trust. In contrast, blockchain ensures transparency by making all transactions visible and immutable across all nodes of the network. This transparency boosts accountability and trust among stakeholders, reducing the risk of fraudulent activities and counterfeit drugs infiltrating the supply chain. Also blockchain's security features are robust compared to relational databases. Relational databases are susceptible to security breaches like SQL injection attacks, which can compromise data integrity and confidentiality. On the other hand, blockchain's decentralized and cryptographic nature makes it highly resistant to unauthorized tampering or data manipulation. The use of cryptographic keys enhances security by ensuring that transactions are validated and recorded securely, minimizing the risk of data breaches and unauthorized access. Hence blockchain's scalability is a significant advantage for pharmaceutical supply chains. Traditional databases often face limitations in scalability due to the exhaustive search space and performance issues beyond certain thresholds. In contrast, blockchain can scale efficiently by extending its network without inherent limitations, accommodating the growing complexity and volume of data in pharmaceutical supply chain operations. The implementation of a blockchain framework, such as the proof of concept (PoC) in Hyperledger Composer, offers tangible benefits in real-life engineering settings. It establishes a transparent and immutable ledger for pharmaceutical product data, enhancing traceability, visibility, and accountability throughout the supply chain. Real-time tracking capabilities enable swift response to quality issues, contamination, or recalls, minimizing financial losses and protecting public health. Additionally, blockchain's decentralized nature reduces reliance on a single authority, fostering trust, compliance, and security across the supply chain ecosystem. Overall, the adoption of blockchain in pharmaceutical product supply chains holds immense potential for optimizing operations, mitigating risks, and ensuring the delivery of safe and authentic medicines to consumers.

The research on implementing a proof-of-concept (PoC) for Supply Chain Risk Management (SCRM) through blockchain technology demonstrates a rigorous approach to assessing feasibility, scalability, and performance. By engaging stakeholders, conducting comprehensive tests across various scenarios, and gathering feedback, the study ensures the validity and reliability of the blockchain-based SCRM system. It provides valuable insights and a roadmap for improving security, efficiency, and transparency in pharmaceutical supply chains.

## Conclusion

The implementation of blockchain technology in pharmaceutical supply chain logistics is a transformative step in reducing the risks associated with loss due to theft, counterfeiting, and diversion. Blockchain's transparent and secure framework instills trust and collaboration among supply chain partners, yielding substantial benefits for businesses and consumers alike. The proposed Supply Chain Risk Management (SCRM) blockchain application, designed for the secure shipping of pharmaceutical raw materials, has proven effective in providing a tamper-proof and auditable trail of all activities by various participants, including suppliers, manufacturers, distributors, and transporters. This robust transparency and trust-building measure are instrumental in ensuring the smooth functioning of the pharmaceutical supply chain. And also the performance evaluation of the SCRM using the Hyperledger Caliper tool demonstrates its scalability, showcasing acceptable latency and throughput even under high transaction volumes. This resilience positions it as a viable solution for automating the raw material shipping industry in the future. With its capacity to handle substantial transaction volumes while maintaining high performance and security, the SCRM blockchain application holds the potential to revolutionize the pharmaceutical raw material shipping sector. It stands as a secure, transparent, and integrated platform for all stakeholders, promising to reduce fraud, enhance efficiency, and elevate the overall quality of the supply chain process.

While this research has unveiled the promising potential of blockchain in pharmaceutical supply chain logistics, it's crucial to acknowledge its limitations. Firstly, the research primarily focuses on raw material shipping, and the findings might not be directly transferable to other segments of the pharmaceutical supply chain. Secondly, real-world implementation challenges, such as regulatory compliance and technology adoption, were not deeply explored in this research. Additionally, the evaluation of the SCRM application's performance, while encouraging, was conducted in a controlled environment and may encounter unforeseen challenges during widespread implementation. So To build on these findings and address the research limitations, future studies should explore the application of blockchain technology across various aspects of pharmaceutical supply chain logistics. Investigating real-world implementation challenges and regulatory compliance issues is essential to gauge the feasibility of scaling up this technology. Additionally, research into the human factors and organizational dynamics impacting the adoption of blockchain in the pharmaceutical supply chain could provide valuable insights.

## Data Availability

The datasets generated and/or analysed during the current study are not publicly available due security reasons but are available from the **First author—Satyabrata Dash** on reasonable request..
